# Isolation, Identification, and Characteristics of *Aeromonas salmonicida* subsp. *masoucida* from Diseased Starry Flounder (*Platichthys stellatus*)

**DOI:** 10.3390/pathogens14030257

**Published:** 2025-03-05

**Authors:** Soo-Ji Woo, So-Sun Kim, Ahran Kim, Mi-Young Cho, Jeong-Wan Do

**Affiliations:** 1 Aquaculture Industry Research Division, East Sea Fisheries Research Institute, National Institute of Fisheries Science, Gangneung 25435, Republic of Korea; wsj2215@korea.kr (S.-J.W.); ssokim81@korea.kr (S.-S.K.); 2 Pathology Division, National Institute of Fisheries Science, Busan 46083, Republic of Korea; arkim110@korea.kr (A.K.); mycho69@korea.kr (M.-Y.C.)

**Keywords:** *Aeromonas salmonicida*, aquaculture, flatfish, pathogen, starry flounder, virulent

## Abstract

*Aeromonas salmonicida* is a predominant pathogen that infects fish. The pathogen *A. salmonicida* subsp. *masoucida* (ASM) was isolated for the first time from diseased starry flounders (*Platichthys stellatus*). Our study aimed to isolate, characterize, and investigate the pathogenicity of ASM. Bacterial species were identified using *16s rRNA*, *gyrB*, *dnaJ*, and *vapA* analyses. Phylogenetic tree analysis revealed that the ASM strains were clustered with the ASM ATCC strain and other strains isolated from black rockfish. In the antimicrobial susceptibility test, the three ASM strains were considered non-wild types for enrofloxacin, florfenicol, flumequine, oxolinic acid, and oxytetracycline susceptibility. Histopathological analysis revealed bacterial colonies in the secondary lamella and heart, indicating that ASM strains are highly virulent in fish. Comparative analysis and annotation via genome sequencing revealed that, among the 1156 factors, adherence factors were the most prevalent putative virulence determinants, followed by the effector delivery system and adherence. ASM was found to possess 43 type III secretion systems, 22 type VI secretion systems, 11 antimicrobial resistance genes, 3 stress genes, and prophage regions. These findings provide new insights into the virulence profile of ASM and highlight the risk posed by emerging pathogenic strains to starry flounders.

## 1. Introduction

The starry flounder (*Platichthys stellatus* Pallas 1787) is a dominant and economically significant flatfish species cultured in various areas, including the eastern coastal regions of Korea. It is a large species widely distributed in low-temperature zones, extending from the north of central Japan to Russia’s Maritime Province, the Otsuk Sea, the Bering Sea, and California Bay in the United States [1]. The annual production capacity of starry flounders is approximately 8000 tons, ranking third (9.7%) in Republic of Korea’s mariculture production and totaling 79,700 tons in 2023 [2]. As a cold-water fish species, starry flounders actively feed at temperatures below 15 °C, with an optimal growth temperature of 13–18 °C [3]. However, fish are susceptible to diseases in summer. Several infectious diseases, including infections caused by *Edwardsiella tarda* [4], *Streptococcus parauberis* [5], red sea bream iridovirus [6], *Trichodina* [7], and *Enteromyxum leei* [8], have been reported in starry flounders.

In April 2023, a disease outbreak occurred across starry flounder farms in Pohang-si, Gyeongsangbuk-do, Korea, with a mortality rate of up to 30%. The water temperature was 13–14 °C. Diseased starry flounders exhibited exophthalmia, hemorrhage of the eyes, jaw, liver, gills covered with mucus, and spleen enlargement. The pathogens were isolated from lesions and identified as *Aeromonas salmonicida* subsp. *masoucida*.

*Aeromonas salmonicida* is the causative agent of furunculosis, a devastating disease characterized by skin ulceration and bleeding. The five subspecies of *Aeromonas salmonicida* subsp. *masoucida* can be divided into typical strains, including *A. salmonicida* subsp. *Salmonicida*, which primarily causes disease in salmonids; and atypical strains, including *A. salmonicida* subsp. *achromogenes*, *masoucida*, *pectinolytica*, and *smithia*, which are mainly isolated from non-salmonid fish [9]. All subspecies, except for *pectinolytica*, have been associated with fish diseases. Epizootic outbreaks of *A. salmonicida* in turbot (*Scophthalmus maximus*) [10,11], halibut (*Hippoglosus hippoglosus*) [12], cod (*Gadus morhua*) [13], crucian carp (*Carassius auratus*) [14], Atlantic salmon (*Salmo salar*) [15], spotted wolffish (*Anarhichas minor*) [16], brook trout (*Salvelinusn fontinalis*) [17], sea bream (*Sparus aurata*) [18], black rockfish (*Sebastes schlegeli*) [19], and red swamp crayfish (*Procambarus clarkii*) [20] have been reported. Recently, the efficacy of vaccines against *A. salmonicida* subsp. *achromogenes*, *Vibrio anguillarum* [21], and *A. salmonicida* subsp. *masoucida* in turbot [22] has been investigated. However, information regarding the molecular identification and pathology of *A. salmonicida* subsp. *masoucida* infections in starry flounders are scarce.

Although several pathogens are known to target starry flounder in the aquatic environment, novel pathogens may emerge due to natural disasters such as climate change. Therefore, epidemiological investigations are needed to identify potential pathogens so that appropriate management and treatment strategies can be employed in a timely manner.

In this study, we isolated, characterized, and investigated the pathogenicity of *A. salmonicida* subsp. *masoucida* infections in starry flounders. We performed phylogenetic tree analysis and an antimicrobial susceptibility test and analyzed the genomic and histopathological features of *A. salmonicida* subsp. *masoucida* in fish. To the best of our knowledge, this is the first report investigating *A. salmonicida* subsp. *masoucida* in starry flounder, and our findings provide a scientific reference for the diagnosis and prevention of the emergence of new pathogenic strains in the Korean aquaculture industry.

## 2. Materials and Methods

### 2.1. Clinical Signs and Sample Collection

**Figure 1:**
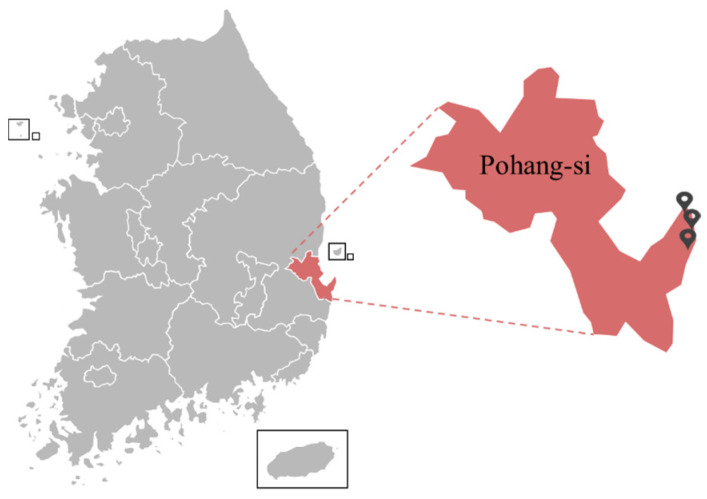
Map showing the location of the starry flounder farms where the epizootic disease outbreak occurred in Korea. The black boxes are islands from Korea.

**Figure 2:**
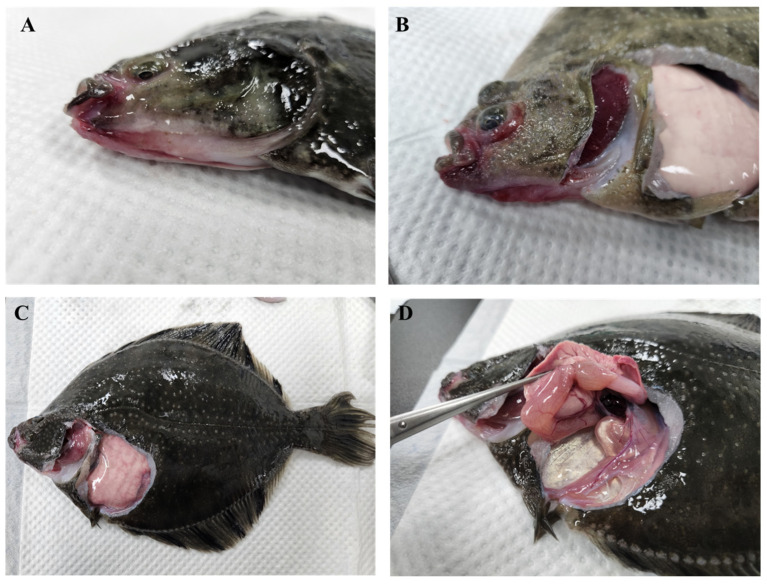
Clinical signs of *Aeromonas salmonicida* subsp. *masoucida* infection in starry flounders. (**A**) Severe hemorrhage of the eyes and jaws; (**B**) exophthalmia and hemorrhages of the gills; (**C**) hemorrhages in the gills; (**D**) spleen enlargement and congestion.

An epizootic disease outbreak occurred at three starry flounder farms (36°02′32.0″ N 129°34′47.6″ E, 35°57′34.2″ N 129°32′55.3″ E, and 36°02′39.8″ N 129°34′53.0″ E) in Pohang-si, Gyeongsangbuk-do, Republic of Korea in April 2023 (Figure 1). Diseased starry flounders (272.8 ± 12 g and 23.8 ± 2 cm) exhibited exophthalmia, hemorrhage of the eyes, jaw, and liver, gills covered with mucus, and spleen enlargement (Figure 2A–D). The diseased fish showed dark color, poor appetite, and abnormal swimming behavior. The water temperature was 13–14 °C, and the mortality rate was 30%. The diseased fish (*n* = 15) were immediately transported to the laboratory in oxygenated bags at 13 °C. For bacterial isolation, diseased fish were euthanized with 150 μg mL^−1^ tricaine methane sulfonate (MS-222, Sigma-Aldrich, St. Louis, MO, USA), in accordance with the guidelines of the Institutional Animal Care and Use Committee of the National Institute of Fisheries Science (approval number: 2023-NIFS-IACUC-30). Lesions (eyes and jaw) and the internal organs (spleen and kidney) were directly streaked on tryptone soybean agar (Difco, Maryland City, MD, USA) with 1% NaCl (*w*/*v*) at 25 °C for 24 h. The bacterial strains are presented in Table S1. The bacterial strains commonly isolated from tissues per fish farm were named ASM1, ASM2, and ASM3 (Table 1). The fish were confirmed to be negative for parasitic and viral infection.

### 2.2. Isolation and Identification of Bacteria

Single colonies were obtained from diseased fish, and genomic DNA was extracted using an AccuPrep^®^ Genomic DNA Extraction Kit (Bioneer, Daejeon, Republic of Korea) following the manufacturer’s instructions. The genomic DNA was stored at –20 °C for further analysis. To determine the specific genera and species of the bacteria, PCR primers targeting *16s rRNA* [23], DNA gyrase subunit B (*gyrB*) [24], and *dnaJ* [25] genes were amplified and sequenced following previously reported methods (Table S2). Virulence array protein A (*vapA*) amplification was carried out using a PRoFlex PCR System (Applied Biosystems, Waltham, MA, USA) with the following conditions: a 5 min initial denaturation step at 94 °C, followed by 35 cycles at 94 °C for 30 s, 54 °C for 30 s, and 75 °C for 30 s, with a final extension step of 7 min at 72 °C. The amplified products were examined using agarose gel electrophoresis (1.2%) and confirmed using sequence analysis (Bionics, Seoul, Republic of Korea). After nucleotide sequence analysis, species identification and similarity calculations were performed to detect homologous sequences in GenBank using BLASTn (https://blast.ncbi.nlm.nih.gov/Blast.cgi, accessed on 4 May 2023). Consensus sequences were aligned using BioEdit [26].

### 2.3. Phylogenetic Analysis

For phylogenetic tree analysis, the *vapA* sequencing results were analyzed using the neighbor-joining (NJ) method, with 1000 bootstrap replications, in the MEGA-X program [27].

### 2.4. Antimicrobial Susceptibility Test

The susceptibility of pathogenic bacteria to antimicrobials was analyzed using a standard broth microdilution method [28]. The minimum inhibitory concentration (MIC) was determined using KRAQ3 and KRAQ4 (Trek Diagnostic System, East Grinstead, UK) plates, as previously described [29]. MICs for amoxicillin (0.06–16 μg mL^−1^), ampicillin (0.5–128 μg mL^−1^), doxycycline (0.12–128 μg mL^−1^), enrofloxacin (0.03–32 μg mL^−1^), erythromycin (0.03–64 μg mL^−1^), florfenicol (0.06–64 μg mL^−1^), flumequine (0.12–128 μg mL^−1^), oxolinic acid (0.5–32 μg mL^−1^), and oxytetracycline (0.12–256 μg mL^−1^) were used. Briefly, the bacteria were cultured on tryptone soybean agar for 24 h at 25 °C, suspended in demineralized water (T3339, Thermo Fisher, Waltham, MA, USA) adjusted to 0.5 McFarland standard, and diluted in cation-adjusted Mueller–Hinton broth (T-3462, Thermo Fisher, USA) to achieve a concentration of 5 × 10^5^ CFU mL^−1^. After incubation at 28 °C for 24 h, the antimicrobial susceptibility was determined by measuring the MIC, and the strains were categorized as wild type (WT) or non-wild type (NWT) according to the epidemiological cut-off value (ECV) studies [30,31].

### 2.5. Whole Genome Sequencing

#### 2.5.1. DNA Sequencing and De Novo Genome Assembly

A DNeasy Blood and Tissue Kit (Qiagen, Hilden, Germany) was used to extract total DNA from the *A. salmonicida* subsp. *masoucida* strain ASM3 following the manufacturer’s instructions. The isolated DNA was sequenced using two different sequencing systems, PacBio Sequel (Pacific Biosciences, Menlo Park, CA, USA) and Novaseq6000 (Illumina, San Diego, CA, USA), which were established using long- and short-read sequencing techniques. Sequencing was performed by DNA Link (Seoul, Republic of Korea) and Insilicogen (Seoul, Republic of Korea), an authorized service provider in Republic of Korea. The Illumina paired-end sequences were initially subjected to the filtering of technical artifacts and adapters using Trimmomatic v. 0.32 [32]. These Illumina reads were used for the error correction of PacBio reads in the CLC Assembly Cell v. 5.1.1 (Qiagen, Hilden, Germany). The corrected reads were used for the initial de novo draft version of the *A. salmonicida* subsp. *masoucida* genome using the FALCON v1.8.1 haplotype assembler [33], and the assembly was polished with Pilon [34]. The assembled contigs were scaffolded to the chromosomal scale using the reference *A. salmonicida* (GCF_028355655.1) and RagTag v 2.1.0 method [35]. The scaffolded contigs were assessed for completeness using BUSCO v5.4.7, with the Viridiplantae_odb10 reference dataset [36].

#### 2.5.2. Genome Size Estimation

All Illumina preprocessed sequences from the paired-end library were subjected to genome size estimation using the *k*-mer method. The *k*-mer frequencies (*k*-mer size = 17) were obtained using Jellyfish v2.1.3 [37], and the genome coverage depth was calculated as: (*k*-mer coverage depth × average read length)/(average read length − *k*-mer size + 1). Genome size was calculated as the total base number/genome coverage depth. The *k*-mer coverage depth was determined using the major peak in the *k*-mer distribution.

#### 2.5.3. Gene Prediction and Annotation

Gene annotation was performed using the Prokka version 1.12b software [38]. Functional annotations were performed using GO [39], KEGG [40], SwissProt [41], and egg-NOG analyses [42]. AMRFinderPlus [43], the comprehensive antibiotic resistance database (CARD), and the virulence factor database (VFDB) [44] were used to identify antimicrobial resistance genes and bacterial virulence factors.

### 2.6. Histopathology

The gills, liver, kidney, spleen, heart, and intestine were collected from diseased starry flounder and fixed in 10% formalin. This was followed by dehydration in a series of ethanol solutions (30%, 70%, 90%, and 100% EtOH). The tissue samples were embedded in paraffin. The paraffin-embedded samples were cut into 5 μm thick sections using a microtome (Leica, Wetzlar, Germany). Subsequently, the samples were stained with hematoxylin and eosin (H&E) and observed under a BX53 light microscope (Olympus, Tokyo, Japan).

## 3. Results

### 3.1. Isolation and Identification of A. salmonicida subsp. masoucida

The clinical signs of diseased starry flounder included severe hemorrhage of the eyes and jaw, gills covered with mucus, and spleen enlargement. *A. salmonicida* subsp. *masoucida* was isolated from affected tissues. Four housekeeping genes (*16s rRNA*, *gyrB*, *dnaJ*, and *vapA*) from three *A. salmonicida* subsp. *masoucida* strains (ASM1, ASM2, and ASM3) isolated from the diseased fish were amplified and sequenced. The obtained sequences were analyzed using the NCBI BLASTn tool. Sequence analysis using *16s rRNA*, *gyrB*, and *dnaJ* identified three strains as *A. salmonicida*, while all strains were identified as *A. salmonicida* subsp. *masoucida* using the *vapA* gene (Table 2).

### 3.2. Phylogenetic Analysis

**Figure 3:**
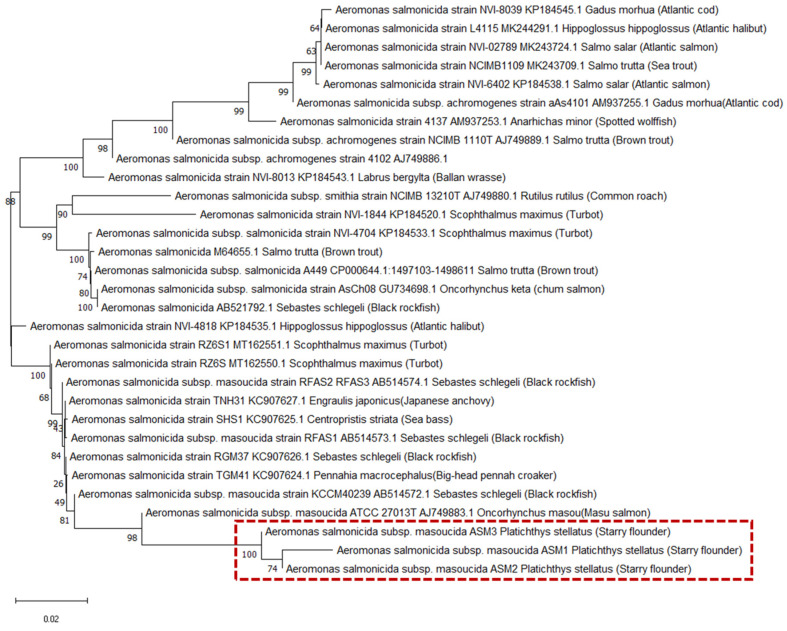
Phylogenetic tree analysis of *Aeromonas* species based on *vapA* sequences. A phylogenetic tree was generated using the neighbor-joining method using MEGA-X software. The numbers next to the branches indicate the percentage values for 1000 bootstrap replicates.

A phylogenetic tree was constructed for the three isolated strains, ASM1, ASM2, ASM3, and the reference *Aeromonas* species (Figure 3). This study used the *vapA* marker because it is difficult to distinguish genetic diversity within *Aeromonas* species using markers specific to other subspecies. Based on the phylogenetic relationship, the three strains were clustered with *A. salmonicida* subsp. *masoucida* ATCC 27013T, which was isolated from masu salmon, confirming their identity as *A. salmonicida* subsp. *masoucida*. Other *Aeromonas* species, including *A. salmonicida*, *A. salmonicida* subsp. *salmonicida*, *A. salmonicida* subsp. *smithia*, and *A. achromogenes,* were clustered in separate branches. The three strains isolated from the starry flounder showed relatively close phylogenetic relationships with the ATCC strain and other strains isolated from black rockfish, indicating a strong homologous correlation between the strains.

### 3.3. Antimicrobial Susceptibility Test

Nine antimicrobial susceptibility tests were performed to examine the three strains (Table 3). The MIC values for amoxicillin and ampicillin were 16< and 128< μg mL^−1^, respectively. The MIC values for enrofloxacin and erythromycin were 1 and 2 μg mL^−1^, respectively. The MIC values for florfenicol ranged from 16 to 64 μg mL^−1^, whereas those for oxytetracycline ranged from 16 to 128 μg mL^−1^. Based on the MIC, the three strains were categorized as WT or NWT according to the ECVs. (Table 3). Applying an ECV of 8 μg mL^−1^ for florfenicol, the three isolates were categorized as NWTs. Consequently, the three strains were categorized as NWTs for enrofloxacin, florfenicol, flumequine, oxolinic acid, and oxytetracycline. However, we could not classify the strains as WT or NWT for amoxicillin, ampicillin, doxycycline, and erythromycin due to the lack of reported ECVs for these antimicrobials in other studies.

### 3.4. Histopathology

**Figure 4:**
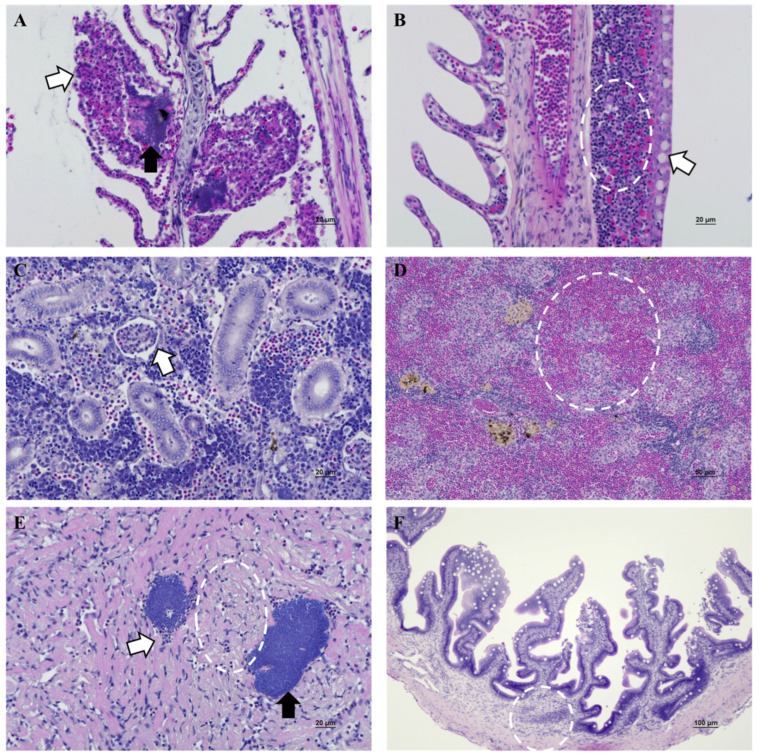
Histopathological changes in the gills (**A**,**B**), kidney (**C**), spleen (**D**), heart (**E**), and intestinal (**F**) tissues of the diseased starry flounders. (**A**) Epithelial hyperplasia (white arrow) and bacterial colonies (black arrow) in the gills, H&E × 400; (**B**) inflammatory cell infiltrates (white circle) and mucous cell hyperplasia (white arrow) in the gills, H&E × 400; (**C**) atrophy and lysis of glomerular tuft (white arrow) in the kidneys. H&E × 400; (**D**) congestion (white circle) in the spleen. H&E × 100; (**E**) focal myocardial degeneration (white circle), inflammatory cell infiltrates (white arrow), and bacterial colonies (black arrow) in the heart. H&E × 400; (**F**) inflammatory cell infiltrates (white circle) in the intestine. H&E × 200.

Histopathological analysis showed significant changes in the gills, kidney, spleen, heart, and intestines of the diseased starry flounders (Table S3). Epithelial hyperplasia, mucous cell hyperplasia, bacterial colonies, and inflammatory cell infiltration were observed in the secondary lamellae (Figure 4A,B). Mucous cell hyperplasia was associated with gills covered in mucus upon external examination. However, the liver appeared normal, and the hepatic sinusoids and hepatopancreas were intact. The kidney showed atrophy and lysis of the glomerular tuft (Figure 4C). However, we did not observe tubular vacuolar degeneration or inflammatory cell infiltration. The spleen was congested in white pulp and red pulp (Figure 4D). Myocardial degeneration, inflammatory cell infiltration, and bacterial colonies were clearly visible in the myocardium (Figure 4E). The intestines showed slight inflammatory cell infiltration of the submucosa (Figure 4F).

### 3.5. Whole Genome Sequencing and Analysis

**Figure 5:**
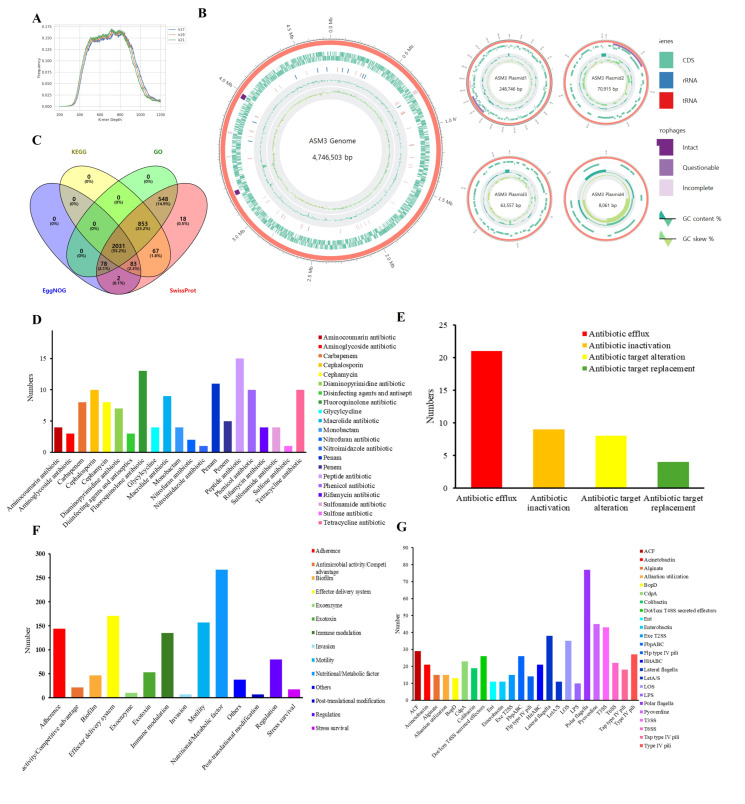
Whole genome atlas of the *Aeromonas salmonicida* subsp. *masoucida* ASM3 strain. (**A**) Distribution of unique *k*-mer depths. The peak of the *k*-mer depth distribution was 17, 19, and 21, respectively; (**B**) genome map of the ASM3 strain and 4 plasmids. Genomic features marked from the outer to the inner circle represent the following: CDS, rRNA, tRNA, prophages, GC content, and GC skew; (**C**) Venn diagram showing annotated functional genes in different databases; (**D**) CARD-annotated drug classification statistics; (**E**) functional annotation determined using the CARD; (**F**) classification of virulence factors annotated using the VFBD; (**G**) subclassification of virulence factors determined using the VFBD.

Whole-genome sequencing was performed to further elucidate the pathogenesis of the strain ASM3, which caused severe pathogenic alterations in the starry flounders. The 17, 19, and 21-mer depth distributions showed a single peak with a flat hill at 700, indicating that the ASM3 strain had very low heterogeneity. The genome size was estimated to range from 5.08 to 5.11 MB based on the *k*-mer analysis results (Figure 5A). The genome of the ASM3 strain was 4,746,503 bp in size, with 58.67% GC content, four plasmids, 123 tRNA genes, and 31 rRNA genes (Table 4) (Figure 5B). The BUSCO scores were 99.54% and 100% complete, indicating high quality. The 5031 coding sequences in the ASM3 genome were compared using GO, KEGG, SwissProt, EggNOG, and Pfam to obtain annotation information related to genome function, which revealed that 2031 proteins corresponding to different functions were predicted by all five databases (Table 5) (Figure 5C). BLASTp analysis of the CARD data revealed 136 genes associated with antibiotic resistance, with the highest number of genes annotated as those conferring resistance to 15 peptide antibiotics, followed by 13 fluoroquinolone antibiotics, 11 penams, and 10 tetracycline antibiotics (Figure 5D). The mechanism of resistance was mainly related to antibiotic efflux and antibiotic inactivation (Figure 5E). BLASTx analysis of the ASM3 genome sequencing data from the VFDB identified 1156 main virulence factors, consisting of 267 nutritional/metabolic factors, 171 effector delivery systems, and 157 motility factors (Figure 5F). Among the genes associated with motility factors, polar flagella-related genes were the most abundant (77 genes). Of the 45 genes associated with the siderophore-scavenging iron gene (pyoverdine), 43 genes were associated with the type III secretion system (*T3SS*), and 22 were associated with the type VI secretion system (*T6SS*) (Figure 5G). ASM3 comprised most of the virulence factors correlated with the *Aeromonas* genus, but *T6SS secreted effectors* were not detected (Table 6). *Colibactin*, *aerolysin*, and *rtxA* were identified as exotoxins. Additionally, the *fur* and *csrA* regulation genes, which originated from *Salmonella* and *Legionella*, respectively, were identified. BLASTx analysis of the ASM3 genome sequencing data from AMRFinderPlus revealed that ASM3 had 11 antimicrobial resistance genes and three stress genes (*arsC*, *arsD*, and *qacEdelta1*) (Table 7). ASM3 had prophage regions with scores of 140 and 150, illustrating intact regions with a length of 32.8 and 35.3 kb, respectively (Table 8).

## 4. Discussion

Domestic consumption of starry flounders has increased by 3.5-fold over the past 10 years due to its wide salinity tolerance and high marketability [2,45]. As the burden of management costs for olive flounders (*Paralichthys olivaceus*) has rapidly increased, starry flounders have been increasingly produced as an alternative breed due to their higher mortality rates [46]. However, the abnormal mortality rate of starry flounders, which are otherwise known for their high disease tolerance, has been reported since 2022, raising concerns among aquaculture farmers. This is believed to be caused by recessive inbreeding and climate change. Loss of genetic diversity and reduced immunity resulting from recessive inbreeding increase vulnerability to pathogens [47]. Climate change has promoted the emergence of new strains of pathogens [48]. For example, *A. salmonicida* subsp. *masoucida*, known to cause infections in other fish species, such as turbot [22,49], black rockfish [19], and red swamp crayfish [20], has recently been recognized as causing increased mortality rates in starry flounders for the first time.

Analysis of *16s rRNA* and various housekeeping genes, including *gyrB*, *rpoB*, *cpn60*, and *dnaJ,* has been used to characterize *Aeromonas* species [50,51]. Although the *16s rRNA* gene is universally considered an efficient tool for identifying bacterial species, its use is controversial because of reading errors when it shows highly conserved regions within the genus *Aeromonas* [52]. *A. trota* and *A. caviae* differ by up to three nucleotides in the *16s rRNA* sequence [53], whereas *A. sobria* and *A. veronii* differ by 12 nucleotides [53]. To overcome the limitations of the high similarity of *16s rRNA*, *gyrB* (which encodes subunit B of DNA gyrase) and *dnaJ* (which encodes heat shock protein 40), were used to differentiate the three strain groups. In this study, the strains ASM1, 2, and 3 were all identified *A. salmonicida* based on the *16s RNA*, *gyrB*, and *dnaJ* genes. A previous report examining the inter- and intraspecific relationships of 53 *Aeromonas* genera based on *gyrB* sequences demonstrated a 2.2% nucleotide substitution rate in *A. salmonicida gyrB* [54]. Additionally, the mean gene divergences of *gyrB* and *dnaJ* in 32 *Aeromonas* strains were 5.2% and 6.8%, respectively, whereas those of *16s rRNA* was 1.4% [51]. Therefore, *gyrB* and *dnaJ* proved to be better identification genes than *16s rRNA* for the characterization of the genus *Aeromonas* after isolation from diseased starry flounders.

*VapA*, which encodes the A-layer surface protein array, serves as both an epizootiological marker and a molecular subtyping marker for *A. salmonicdia* subspecies, with *vapA* types ranging from 1 to 23 [55]. The pathogenic strain isolated from turbot with skin ulcers was identified as *A. salmonicida* subsp. *masoucida* based on the *vapA* gene sequence [56]. In contrast, a pathogenic *A. salmonicida* strain isolated from snakehead was reported to lack *vapA*, as confirmed by *vapA* gene cloning [57]. Based on the results of the phylogenetic analysis, ASM 1, 2, and 3 were identified as *A. salmonicdia* subsp. *masoucida*, based on *vapA* gene expression and their clustering with *A. salmonicida* subsp. *masoucida* ATCC 27013T. This finding aligns with previous studies that assigned the ATCC 27013 type strain as A-layer type 7 [55]. Most A-layer types can be linked to a specific host with only one or a few *vapA* clusters [55]. While most A-layers in types 1 to 7 originate from the Norwegian coast, type 7 is found in the Pacific Ocean and Asia, suggesting regional specificity [58]. It is necessary to obtain more *A. salmonicdia* subsp. *masoucida* strains to perform clustering analysis with various host fish species and to identify the geographic distribution of these hosts.

With the growing issue of antibiotic resistance in aquaculture, multi-drug resistance in *Aeromonas* poses a serious public health problem [29]. The only way to reduce antimicrobial resistance is through the use of commercial vaccines; however, to date, no vaccine exists for *A. salmonicdia* subsp. *masoucida* in starry flounder farming in the Republic of Korea, except for *S. parauberis* vaccines, such as PRO-VAC^TM^ S PARA. In our study, we found that all strains were categorized as NWT for enrofloxacin, florfenicol, flumequine, oxolinic acid, and oxytetracycline resistance, indicating multi-drug resistance. Although no ECV criteria have been published for ampicillin and amoxicillin, the high MIC values of 16< and 128< are consistent with the characteristic of the *Aeromonas* genus, which is innately resistant to β-lactams [59]. Specifically, the ASM3 strain harbored antimicrobial resistance genes, including *ampC*, *blaOXA*, and *blaOXA,* which are related to resistance to the β-lactam class. Furthermore, the presence of the *catB3*, *catA2*, and *floR* genes in the ASM3 strain contributes to high MIC values of florfenicol at 64 μg mL^−1^. *FloR* has been identified either on the chromosomes or plasmids of Gram-negative bacteria and is associated with mobile genetic elements and genomic islands [60]. Notably, *A. salmonicida* subsp. *masoucida* may be considered an agent of antimicrobial resistance dissemination from aquaculture to the natural environment.

Histopathological analysis revealed that the diseased starry flounder exhibited inflammatory cell infiltration of the gills with epithelial hyperplasia and fusion of secondary lamellae. Gills covered with mucus may occur because the gills serve as the first physical defense against bacterial invasion [61]. This indicates that mucus viscoelasticity determines its ability to block many types of motile bacteria [62]. Additionally, we observed bacterial colonies in the gills and heart, indicating that the bacteria circulated in the blood–vascular system. This was consistent with the immunohistochemistry results for *A. salmonicida* subsp. *salmonicida* in challenged turbots, which showed a strong positive reaction in the lumen of the blood vessels of the secondary lamellae and heart [63]. Our results align with the infection route, indicating that the gills, intestine, and skin, as mucosal immune-related tissues, are the main infection sites of *A. salmonicida* subsp. *masoucida* with changes in the bacterial amount [64]. Therefore, it is assumed that the bacteria enter the bloodstream after colonization and are then transmitted to internal tissues, resulting in septicemia with mass mortality. Inflammatory cell infiltration of the mucosal layer indicates that intestinal epithelial cells target pathogens by producing inflammatory cytokines [65]. TNF-α, IL-1β, and IL-8 mRNA expression were significantly increased in the intestine after infection with *A. hydrophila* from grass carp [66]. Skin nodules are the atypical representative symptoms of *A. salmonicida* [67], and we found that the main clinical signs were hemorrhages around the jaw and mouth. Although a few previous studies have presented findings that were directly comparable with our results, granulomatous dermatitis was prominent in turbot challenged with *A. salmonicida* subsp. *masoucida*, indicating that it may facilitate infection by pathogens [22,49]. Therefore, *A. salmonicida* subsp. *masoucida* may present different clinical signs according to the host species specificity. The importance of *A. salmonicida* subsp. *masoucida* as a fish pathogen has been previously confirmed.

CARD analysis demonstrated that genes responsible for resistance to peptide antibiotics constituted to the highest proportion of antibiotic resistance genes, but it was difficult to compare the MIC results owing to the lack of MIC data for peptide antibiotics. In addition, peptide antibiotics, including colistin and bacitracin, have only been approved for use in cattle, pigs, and chickens and not for fish in the Republic of Korea. The possession of resistance genes for unapproved antibiotics indicates the emergence of resistant bacteria in aquaculture and potential horizontal transmission between livestock and humans. Similarly, the transposon of a 67-kb plasmid of *A. salmonicida* subsp. *salmonicida* carried the *catB3* gene, which aligns with the findings of our study and suggests the possibility of transfer through conjugation to *A. hydrophila* [68]. The *Aeromonas* genus includes 4 major and 14 minor categories of virulence factors, as identified in the VFDB database. Analysis of whole genome sequencing data revealed 1156 virulence factors coding for nutritional/metabolic factors, effector delivery systems, and exotoxins. We found that the *T3SS* and *T6SS* genes, which encode the secretion system, contribute to the virulence. *A. hydrophila* possesses a *T3SS* that delivers four effector proteins to target host cells, whereas *T6SS*-associated virulence factors play a role in the secretory apparatus, promoting bacterial virulence [69]. The genomes of 105 *Aeromonas* strains isolated from environmental or pathogenic sources were used to identify the distribution and cytotoxicity of 21 *T3SS* effector families [70]. The deletion of two genes (*hcp1* and *vgrG1*) encoding type VI secretion system proteins of the *T6SS* in virulent *A. hydrophila* resulted in a 2.24-fold reduction in virulence when tested in catfish fingerlings [71]. *Aerolysin*, a hemolytic toxin, was detected in our study, as well as in *A. hydrophila* [72] and *A. veronii* [73]. Additionally, the isolates harbored the *rtxA* gene, an exotoxin-encoding lysine acyltransferase. *rtxA* is restricted to the phylogroups Hydrophila and Salmonicidia, including *A. salmonicida* subsp. *pectinolytica*, among 65 *Aeromonas* strains [74]. Therefore, our findings suggest that genomic detection of these virulence genes may help identify targets for developing new vaccines against this emerging pathogen. Phages are viruses that infect bacterial cells, disrupt bacterial metabolism, and cause bacterial lysis [75]. In the present study, ASM3 exhibited two intact prophage regions. Most phages of *A. salmonicida* were classified as those belonging to the *Myoviridae* family and are more prevalent than other phages [76]. In particular, modification of the receptor, including lipid A of the lipopolysaccharide and the A layer of the outer membrane protein, has been reported as the main mechanism of resistance to phages of *A. salmonicida* [77]. Bacterial mobile elements, such as plasmids, prophages, transposons, and insertion sequences, can be transmitted vertically with cell division or horizontal transfer. Mobile elements are known to potentiate gene gain and loss, contributing to genetic adaptation to new environments and the emergence of a bacterial population [78]. The prophage region present in ASM3 strains is supposed to contribute to its adaptation to a new host, like starry flounders. Further studies are needed to evaluate the potential of *A. salmonicida* subsp. *masoucida* in the treatment and prevention of phage *A. salmonicida* subsp. *masoucida*.

In summary, *A. salmonicida* subsp. *masoucida*, a newly discovered pathogenic *Aeromonas* species, is currently pathogenic to starry flounders. Future research should aim to expand testing to investigate the *A. salmonicida* subsp. *masoucida* virulence characteristics through challenge experiments to determine its full host susceptibility. Studies examining the control of pathogenic factors unique to *A. salmonicida* subsp. *masoucida* should also be performed to understand the main causes of infection in starry flounders. Multifaceted research will enable a better understanding of host susceptibility, assessment of host risks, and the development of vaccines to prevent diseases.

## 5. Conclusions

In conclusion, three pathogenic *A. salmonicida* subsp. *masoucida* strains were isolated for the first time from diseased starry flounders in the Republic of Korea. The bacterial species were identified via analyses of *16s rRNA*, *gyrB*, *dnaJ*, and *vapA*. Phylogenetic analysis revealed that the three ASM strains were clustered with the *A. salmonicida* subsp. *masoucida* ATCC strain. The three strains were categorized as NWT with five antimicrobials, and bacterial colonies were detected in the secondary lamellae and heart. ASM3 genome analysis showed a range of virulence factors, including nutritional/metabolic factors, effector delivery systems, 11 antimicrobial resistance genes, three stress genes, and prophage regions. Future research should investigate the characteristics of *A. salmonicida* subsp. *masoucida* virulence via challenge experiments to determine host susceptibility and elucidate infection mechanisms. This study will support the development of vaccines to prevent disease in starry flounders.

## Figures and Tables

**Table 1:** Information of the sample sources.

Time	Location	Species	Fish	Organs	Strain No.
2023.4.	Pohang-si, Republic of Korea	*A. salmonicida* subsp. *masoucida*	Starry flounder	Kidney	ASM1
2023.4.	Pohang-si, Republic of Korea	*A. salmonicida* subsp. *masoucida*	Starry flounder	Spleen	ASM2
2023.4.	Pohang-si, Republic of Korea	*A. salmonicida* subsp. *masoucida*	Starry flounder	Jaw	ASM3

**Table 2:** Comparison of *Aeromonas salmonicida* subsp. *masoucida* isolates using *16s rRNA*, *gyrB*, *dnaJ*, and *vapA* genes.

Strain	*16s rRNA*	*gyrB*	*dnaJ*	*vapA*
ASM1	*A. salmonicida*	*A. salmonicida*	*A. salmonicida*	*A. salmonicida* subsp. *masoucida*
ASM2	*A. salmonicida*	*A. salmonicida*	*A. salmonicida*	*A. salmonicida* subsp. *masoucida*
ASM3	*A. salmonicida*	*A. salmonicida*	*A. salmonicida*	*A. salmonicida* subsp. *masoucida*

**Table 3:** MIC distribution and ECVs of antimicrobial agents for *Aeromonas salmonicida* subsp. *masoucida* isolates.

Antimicrobials	MIC Distribution (μg mL^−1^)			ECV (μg mL^−1^)	NWT (%)	WT (%)	Reference
	ASM1	ASM2	ASM3				
Amoxicillin	16<	16<	16<	ND	-	-	
Ampicillin	128<	128<	128<	ND	-	-	
Doxycycline	1	2	1	ND	-	-	
Enrofloxacin	1	1	1	≥0.062	100	0	[30]
Erythromycin	2	2	2	ND	-	-	
Florfenicol	16	64	64	≥8	100	0	[31]
Flumequine	4	4	8	≥0.062	100	0	[30]
Oxolinic acid	8	8	16	≥0.25	100	0	[31]
Oxytetracycline	16	128	16	≥2	100	0	[31]

ECV, epidemiological cut-off value; MIC, minimum inhibitory concentration; ND, not possible to determine the ECV; NWT, non-wild type; WT, wild type.

**Table 4:** Key genome features of the *Aeromonas salmonicida* subsp. *masoucida* ASM3 strain.

Features	Value
Genome size (bp)	4,746,503
GC content (%)	58.67
N content (%)	0
Number of contigs	1
Plasmids	4
CDS	4876
Total genes	5031
Protein-coding genes	4876
rRNA genes	31
tRNA genes	123
tmRNA(transfer-messenger RNA, SsrA)	1
BUSCO (proteobacteria_odb10)	C: 99.54%, F: 0.46%, M: 0%
BUSCO (gammaproteobacteria_odb10)	C: 100%, F: 0%, M: 0%

**Table 5:** Functional annotation of protein-coding genes.

DB	CDS	Percent
No Hits	127	(2.52%)
GO	3510	(69.77%)
KEGG	3304	(60.31%)
SwissProt	3680	(73.15%)
EggNOG	2194	(43.61%)
Pfam	0	(0%)
Total	5031	(100%)

**Table 6:** Virulence factor annotation of protein-coding genes in the *Aeromonas salmonicida* subsp. *masoucida* ASM3.

Genus	VFDB		# of Proteins
	Category 1	Category 2	ASM3
*Aeromonas*	Adherence (VFC0001)	*Type IV pili* (VF0082)	27
		*Tap type IV pili* (VF0475)	18
		*Flp type IV pili* (VF0476)	14
		*MAM7* (VF0512)	12
		*MSHA type IV pili* (VF0477)	10
	Biofilm (VFC0271)	*Alginate* (VF0091)	15
		*BopD* (VF0362)	13
	Effector delivery system (VFC0086)	*T3SS* (VF0479)	43
		*Dot/Icm T4SS secreted effectors* (VF0798)	26
		*T6SS* (VF0480)	22
		*Exe T2SS* (VF0478)	15
		*T6SS secreted effectors* (VF0651)	ND
	Exotoxin (VFC0235)	*Colibactin* (VF0573)	19
		*Aerolysin* (VF0481)	3
		*RtxA* (VF0482)	2
	Immune modulation (VFC0258)	*LOS* (VF0044)	35
		*LPS* (VF0171)	10
	Motility (VFC0204)	*Polar flagella* (VF0473)	77
		*Lateral flagella* (VF0474)	38
	Nutritional/metabolic factor (VFC0272)	*Pyoverdine* (VF0094)	45
		*FbpABC* (VF0272)	26
		*Acinetobactin* (VF0467)	21
		*HitABC* (VF0268)	21
		*Allantion utilization* (VF0572)	15
		*Ent* (VF0562)	11
		*Enterobactin* (VF0228)	11
	Others (VFC0346)	*ACF* (VF0127)	29
	Regulation (VFC0301)	*CdpA* (VF0432)	23
		*LetA/S* (VF0262)	11
*Salmonella*		*Fur* (VF0113)	1
*Legionella*		*CsrA* (VF0261)	1

#, The numbers of the proteins.

**Table 7:** Antimicrobial resistance and stress factor annotation of protein-coding genes in the *Aeromonas salmonicida* subsp. *masoucida* ASM3.

Protein ID	Start–End (Strand)	Gene Symbol	Element Type	Cov (%)	ID (%)	Accession
KFIIMLHA_00514	524,007–524,624 (+)	*cphA*	AMR	75.98	97.93	WP_063865210.1
KFIIMLHA_01148	1,162,385–1,163,533 (+)	*ampC*	AMR	100	84.55	WP_096807445.1
KFIIMLHA_03965	4,109,601–4,110,032 (−)	*arsC*	STRESS (METAL)	99.29	76.43	BAA24824.1
KFIIMLHA_03968	4,113,077–4,113,520 (−)	*arsD*	STRESS (METAL)	100	43.54	AAA93060.1
KFIIMLHA_04588	4,725,375–4,726,169 (+)	*blaOXA*	AMR	100	99.62	WP_021139936.1
NA	152,547–153,743 (−)	*tet(A)*	AMR	100	100	WP_000804064.1
NA	160,003–160,554 (+)	*aac(6′)-Ib-cr5*	AMR	100	100	WP_063840321.1
KFIIMLHA_04774	160,688–161,518 (+)	*blaOXA-1*	AMR	100	100	WP_001334766.1
KFIIMLHA_04775	161,656–162,288 (+)	*catB3*	AMR	100	100	WP_000186237.1
KFIIMLHA_04778	164,046–164,687 (−)	*catA2*	AMR	100	100	WP_011751353.1
NA	167,154–167,603 (+)	*arr-3*	AMR	100	100	WP_001749986.1
NA	167,829–168,173 (+)	*qacEdelta1*	STRESS(BIOCIDE)	100	100	WP_000679427.1
KFIIMLHA_04781	168,170–169,009 (+)	*sul1*	AMR	100	100	WP_000259031.1
KFIIMLHA_04800	191,799–193,013 (−)	*floR*	AMR	100	96.29	WP_001747811.1

+ strand, top(forward) strand; −strand, bottom(reverse) strand.

**Table 8:** Bacterial genomic prophage regions of the *Aeromonas salmonicida* subsp. *masoucida* ASM3 strain.

Contig	Length (kb)	Completeness (Score) *	Protein	Start	End	GC%
Contig 1	26.4	Incomplete (30)	10	718,565	744,983	56.42
	6.2	Incomplete (40)	8	2,824,640	2,830,917	54.57
	32.8	Intact (140)	43	3,226,107	3,259,002	60.15
	6.7	Incomplete (30)	7	3,510,611	3,517,403	58.81
	13.6	Incomplete (40)	11	3,685,902	3,699,589	54.70
	35.3	Intact (150)	53	3,957,865	3,993,394	59.30
Contig 2	19.9	Questionable (70)	18	152,528	172,508	56.54
	8	Incomplete (30)	8	180,622	188,720	55.96
Contig 3	11	Questionable (70)	10	1976	13,006	53.12

* Intact (score > 90); questionable (score 70–90); incomplete (score < 70).

## Data Availability

The data presented in this study are available upon request from the corresponding author. The data used to support the findings of this study are included within the article.

## Supplementary Materials

The following supporting information can be downloaded at https://www.mdpi.com/article/10.3390/pathogens14030257/s1: Table S1: The bacterial strains collected from diseased starry flounder; Table S2: PCR primers and their target genes and amplified DNA fragments; Table S3: Scoring of histopathological alterations in the gills, kidney, spleen, heart, and intestine of *Aeromonas salmonicida* subsp. *masoucida*-infected starry flounder.
