# Supplementary material for: Isolation, Identification, and Characteristics of Aeromonas salmonicida subsp. masoucida from Diseased Starry Flounder (Platichthys stellatus)
Source: Pathogens. 2025 Mar 5;14(3):257. doi: 10.3390/pathogens14030257 (PMC11945087; doi:10.3390/pathogens14030257)
Supplement: Supplementary file 1 [file pathogens-14-00257-s001.zip › Table S2.pdf]

Table S2. PCR primers and their target genes and amplified DNA fragments.

Target gene	Primer sequence (5′-3′)	Product size (bp)	Annealing Tm (℃)	Reference
16s rRNA	27F: AGA GTT TGA TCC TGG CTC AG<br>1492R: GGT TAC CTT GTT ACG ACT T	1500	55	[23]
gyrB	gyrB_F: TCCGGCGGTCTGCACGGCGT<br>gyrB_R: TTGTCCGGGTTGTACTCGTC	1100	60	[24]
dnaJ	dnaJ_F: CGAGATCAAGAAGGCGTACAA G<br>dnaJ_R: CACCACCTTGCACATCAGATC	855	60	[25]
vapA	vapA_F: TGAAGGCCAATTCAACGACG<br>vapA_R: CTTGAGAACCGGAGGTCAGC	308	60	This study
