# Supplementary material for: Isolation, Identification, and Characteristics of Aeromonas salmonicida subsp. masoucida from Diseased Starry Flounder (Platichthys stellatus)
Source: Pathogens. 2025 Mar 5;14(3):257. doi: 10.3390/pathogens14030257 (PMC11945087; doi:10.3390/pathogens14030257)
Supplement: Supplementary file 1 [file pathogens-14-00257-s001.zip › Table S3.pdf]

Table S3. Scoring of histopathological alterations in the gills, kidney, spleen, heart, and intestine of the *Aeromonas salmonicida subsp. masoucida* infected starry flounder.

Tissue	Alterations	Strain		
		ASM1	ASM2	ASM3
Gill	Epithelial hyperplasia	1	1	3
	Mucous cell hyperplasia	0	2	1
	Chloride cell hyperplasia	0	1	0
	Telangiectasis	1	2	3
	Fusion of secondary lamellae	1	2	3
	Cellular degeneration & necrosis	2	3	3
	Inflammatory cell infiltrate	2	3	3
	Bacterial colonies	0	3	3
Kidney	Atrophy and lysis of glomerular tuft	3	2	3
Spleen	Spleen congestion	0	1	2
Heart	Myocardial degeneration	1	1	2
Heart Intestine	Inflammatory cell infiltrates	1	1	2
	Bacterial colonies	2	2	3
	Inflammatory cell infiltrates	1	0	1

* Scoring of alteration, 1=mild; 2=moderate; 3=severe
